# Sequential Genome Editing and Induced Excision of the Transgene in *N. tabacum* BY2 Cells

**DOI:** 10.3389/fpls.2020.607174

**Published:** 2020-11-25

**Authors:** Maor Sheva, Uri Hanania, Tami Ariel, Albina Turbovski, Vishal Kumar Rameshchandra Rathod, Dina Oz, Yoram Tekoah, Yoseph Shaaltiel

**Affiliations:** Protalix Biotherapeutics, Carmiel, Israel

**Keywords:** CRISPR/Cas9, genome editing, glyco-engineering, *N. tabacum* BY2 cells, plant glycans, transgene-free, plant biotechnology, recombinant proteins

## Abstract

While plant cells in suspension are becoming a popular platform for expressing biotherapeutic proteins, the need to pre-engineer these cells to better comply with their role as host cell lines is emerging. Heterologous DNA and selectable markers are used for transformation and genome editing designated to produce improved host cell lines for overexpression of recombinant proteins. The removal of these heterologous DNA and selectable markers, no longer needed, can be beneficial since they limit additional gene stacking in subsequent transformations and may pose excessive metabolic burden on the cell machinery. In this study we developed an innovative stepwise methodology in which the CRISPR-Cas9 is used sequentially to target genome editing, followed by its own excision. The first step included a stable insertion of a CRISPR-Cas9 cassette, targeted to knockout the β(1,2)-xylosyltranferase (XylT) and the α(1,3)-fucosyltransferase (FucT) genes in *Nicotiana tabacum* L. cv Bright Yellow 2 (BY2) cell suspension. The second step included the excision of the inserted cassette of 14.3 kbp by induction of specific sgRNA designed to target the T-DNA boundaries. The genome editing step and the transgene removal step are achieved in one transformation run. This mechanism enables CRISPR genome editing and subsequently eliminating the introduced transgenes thus freeing the cells from foreign DNA no longer needed.

## Introduction

Using plant cell suspensions for the production of biotherapeutic proteins is considered a promising direction to overcome some of the limitations of using whole plants as the production platform (Tekoah et al., 2015; Zagorskayaa and Deinekoa, 2017). The plant cell platform is comparable to the industry common practices of using mammalian, bacterial or yeast cells and, unlike whole plants, can be maintained with full compliance with GMP production systems.

The CRISPR-Cas9, Cas12a (also known as Cpf1) and, recently discovered, CasX have proven to be highly efficient valuable tools for precise genome editing across a wide range of cell types and organisms. The CRISPR-Cas system is becoming an increasingly important tool for a wide range of targeted mutagenesis, gene replacement, and other novel applications (Shan et al., 2013; Ma et al., 2015; Filler Hayut et al., 2016; Koonin et al., 2017; Dahan-Meir et al., 2018; Kumlehn et al., 2018; Liu et al., 2019). However, such approaches might result in a transgenic cell line that harbors a long list of various transgenes, some of which are no longer required once they have accomplished their task.

In order to intervene in the glycosylation machinery and to humanize plant produced recombinant biotherapeutics, the CRISPR system was used to engineer the genome of BY2 host cells and *Nicotiana benthamiana* host plants (Hanania et al., 2017; Mercx et al., 2017; Jansing et al., 2019). The presence of selectable markers and nuclease genes (e.g., CRISPR-Cas9) that were used for the targeted mutations, limit their further application in subsequent transformations and may pose excessive metabolic burden on the cell’s machinery. Therefore, the removal of these genes is highly desirable.

To date, many approaches have been developed in whole plants, which segregate sexually, to eliminate the selectable marker that has been stably integrated into the plant genome. These methods include: (1) repeated back crosses and segregation (Zhang et al., 2014; Gao et al., 2016; Char et al., 2017); (2) Co-transformation using two constructs, one of which contains the selectable marker while the other harbors the desired gene of interest, thus allowing for subsequent removal of the selectable gene by genetic segregation (Komari et al., 1996); (3) Homologous recombination between direct repeats using the Cre recombinase microbial enzyme (Dale and Ow, 1991; Wang et al., 2005) or using the yeast FLP recombinases (Davies et al., 1999); and (4) transposable element-based systems (Yoder and Goldsbrough, 1994).

Additional approaches to avoid Cas9 integration within whole plant genome include the following: (1) Transient expression of Cas9 (Zhang et al., 2016; Chen et al., 2018); (2) Transfection of preassembled complexes of purified Cas9 protein and guide RNA (RNP) into plant protoplasts (Woo et al., 2015); (3) Using “suicide” transgenes, such as the *Barnase* gene under the control of the rice REG2 promoter, that effectively kill all of the CRISPR-Cas9 containing pollen and embryos, assuring that any viable embryos will be free of foreign DNA (He et al., 2018); or (4) Coupling the CRISPR construct with an RNA interference element, which targets a herbicide resistance enzyme in rice (Lu et al., 2017), resulting in transgene-free mutated plants.

Unfortunately, besides being highly laborious and time consuming, most of these approaches are not applicable to cells in suspension, due to the asexual nature of propagation of these cells. Thus an approach, specifically targeted at removal of heterologous DNA inserts from plant cells in culture, is of need.

Previous experiments showed procedures for large chromosomal excision in non-plant species (Xiao et al., 2013; He et al., 2014; Zhang et al., 2015; Hao et al., 2016) and large chromosomal excision in plant species (Zhou et al., 2014; Ordon et al., 2017; Cai et al., 2018; Li et al., 2019). Based on these studies, a more effective approach was developed for the removal of the CRISPR-Cas9 construct from the cell’s genome, after the targeted genomic modifications have been accomplished. For this, an innovative methodology was developed that inductively self-excises the entire CRISPR-Cas9/gRNAs harboring construct from the modified transgenic BY2 cells, after the targeted mutations were accomplished.

## Materials and Methods

### Plant Cell Suspensions

*Nicotiana tabacum* cv. BY2 cells (Nagata, 2004) were cultured as a suspension culture in liquid MS-BY2 medium (Nagata and Kumagai, 1999) at 25°C with constant agitation on an orbital shaker (85 r.p.m.). The suspensions were grown at 50 mL of volume in 250 mL erlenmeyers and were sub-cultured weekly at 2.5% (v/v) concentration.

### Construction of the Self-Removed Binary Vector

**FIGURE 1:**
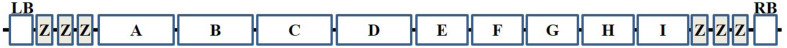
Schematic description of the “self-removable” CRISPR-Cas9 vector used for the transformation of the BY2 cells. LB, left border; ZZZ, three repeats of 23 nucleotides each used as a target for sgRNA-Z; Cassette A, (NosP-HptII-NosT); Cassette B, (35SP-hCas9-OcST); Cassette C, (35SP-codA-OcST); Cassette D, (HSP-sgRNA-Z-OcST); Cassette E, (U6-sgRNA1-5xT); Cassette F, (U6-sgRNA2-5xT); Cassette G, (U6-sgRNA3-5xT); Cassette H, (U6-sgRNA4-5xT); Cassette I, (U6-sgRNA5-5xT); RB, right border; NosP, Nopaline synthase promoter; HptII, Hygromycin phosphotransferase II; NosT, Nopaline synthase terminator; 35SP, 35S cauliflower mosaic virus promoter with omega enhancer; hCas9, human-optimized Cas9 with the SV40 nuclear localization signal; OcST, Octopinesynthase terminator; coda, Cytosine deaminase (negative selectable marker); HSP, heat shock promoter (HSP 18.2) from Arabidopsis thaliana; U6, Arabidopsis U6 promoter; sgRNA-Z, Chimera of crRNA directed to the Z sequences at the boundaries of the vector with tracrRNA; sgRNA 1-5, five chimeras of the five crRNAs with tracrRNA, that were used to knock out *FucT* and *XylT* genes (Hanania et al., 2017); and 5xT, terminator, five repeats of T.

To construct the self-removed binary vector (Figure 1), we used the pBIN19 backbone containing the human codon optimized Cas9 cassette and five cassettes of U6-sgRNAs directed to the *FucT* and *XylT* genes (described earlier, Hanania et al., 2017). The T-DNA was bordered (downstream to the LB and upstream to the RB) by three repeats of Z sequence that were commercially synthesized by Genscript (NJ, United States) and contained three additional cassettes. The first cassette contained a *hygromycin phosphotransferase* gene placed downstream of the nopaline synthase promoter and upstream of the nopaline synthase terminator, used for selection of transformed lines. The second cassette contained a codA gene placed downstream of the 35S promoter and upstream of the octopine synthase terminator, used for selection of the self-removed cells. The third cassette, containing the sgRNA-Z that was commercially synthesized by Genscript (NJ, United States) and placed downstream of the 18.2 Arabidopsis heat shock promoter (HSP) and upstream of the octopine synthase terminator. HSP was used to induce the expression of the sgRNA-ZZZ and in turn the T-DNA excision.

### Transformation of BY2 Cells and Selection of Lines

The final vector was used to transform the tobacco BY2 cells via the Agrobacterium plant transformation procedure (An, 1985). Once a stable transgenic cell suspension was established, it was used for isolating and screening individual cell lines (clones).

Establishing of individual cell lines was conducted by using highly diluted aliquots of the transgenic cell suspension and spreading them on solid medium. The cells were allowed to grow until small calli (plant cell mass) developed. Each callus, representing a single clone, was then re-suspended in liquid medium and sampled.

### Screening of the XylT/FucT Knocked-Out Lines and Testing the Re-Cloned Lines

Individual transformed lines were screened using an ELISA test for the absence of Fucose and Xylose containing glycans as described previously (Hanania et al., 2017). Lines (28, 76, and 90) that were assumed to be knocked out for the *FucT* or *XylT* genes at the first screening stage, were then analyzed by SDS-PAGE and western blot using anti-HRP antibodies (Agrisera AS09-549, Vännäs, Sweden), which specifically recognize both xylose and fucose moieties on the plant specific glyco-proteins (as described in Hanania et al., 2017). For further analysis, anti-Cas9 (Sigma-Aldrich MAC133, Rehovot, Israel), anti-codA antibodies (custom prepared by Genscript, NJ, United States) and anti-HptII antibodies (Artron Bio research A85-Ab1, Philadelphia, United States) were used.

### Total Protein Extraction

A total of 100 mg of cells were transferred into 2 mL tube containing one glass bead (5 mm). A volume of 200 μL sample buffer (100 mM Tris HCL buffer pH 6.8, 4% SDS, 0.5 M DTT, 30% glycerol and 0.4 mg/mL of bromophenol blue) were added and the sample was tissue-lysed for 10 min at 28 Hz using TissueLyser II (Qiagen, Hilden, Germany). The sample was boiled for 10 min. and centrifuged at 20,000 *g* for 10 min. and supernatant was transferred into a new tube.

### PCR Reactions

PCR was performed using forward and reverse primers with 35 cycles of the following procedure: 95°C for 1 min, 60°C for 20 s, and 72°C for 1 min. The PCR products were separated by electrophoresis on an ethidium bromide-stained 1% agarose gel.

### Heat Induction and Selection on Medium Supplemented With 5-FC

To induce the expression of the sgRNA-ZZZ in the XylT/FucT, knockout BY2 cell lines 28, 76, and 90, the cultures were repeatedly (twice weekly for 2 consecutive week) exposed to heat treatment (2.5 h at 37°C) in a water bath. After induction and for the selection of cells in which the transgene was excised, the cells were transferred to selection medium containing 750 mg/L 5-Flurocytosine (5-FC) while the hygromycin (that served as a selection agent in the previous step) was omitted.

### Analysis of Genome Modifications of the Knocked-Out Cell Lines

Genomic DNA was extracted using the DNeasy plant mini kit (Qiagen, Hilden, Germany). The DNA was amplified by PCR using specific primers for *XylT* and *FucT* genes (Supplementary Table 3). The PCR products were sub-cloned into the pGEMT vector. Colonies were sequenced and were aligned with the wild-type target sequences to determine the mutations.

### Southern Blot Analysis

Total genomic DNA for southern blot analysis was isolated from BY2 cells based on Shure et al. (1983) with additional steps (Supplementary Method). Digested DNA was then separated on 0.8% agarose gel electrophoresis system at 100 V for 5 h in 1 × TAE. Separated DNA was then capillary transferred to a nylon membrane and immobilized by UV-crosslinking using Stratalinker (Stratagene, La Jolla, CA, United States). Hybridization was done using DIG probes. Signal intensity was scanned with high resolution chemiluminescence settings using a ChemiDoc Touch Imaging System (Bio Rad, Herculaes, CA, United States) with Image Lab Software ver 5.2.1 (Bio Rad, Herculaes, CA, United States).

## Results

### Construction of the “Self-Removable” CRISPR-Cas9 Vector

In a previous publication (Hanania et al., 2017), we reported the construction of a vector for stable transformation, which was designed to drive the constitutive expression of Cas9 together with five sets of sgRNAs targeted to the *N. tabacum* genome, which are known to contain two XylT genes (Ntab-XylT A and B) and five FucT genes (Ntab-FucT A, B, C, D, and E). To enable its own self-excision and the subsequent selection of “transgene-free” cell lines thereafter, the previously described vector (Hanania et al., 2017) was further supplemented with the following components: (1) three repeats of 23 nucleotides each, designated as “ZZZ,” which were framed on its two edges; (2) an additional g-RNA-Z driven by the heat inducible HSP18.2 promoter (Takahashi and Komeda, 1989); and (3) a cytosine deaminase (codA) negative selectable marker (de Oliveira et al., 2015) driven by a 35S promoter (Figure 1 and Supplementary Table 1).

The three 23 nucleotide repeats, which do not exist on the *N. tabacum* genome, were designed to serve as targets for the Cas9-sgRNA-Z. The heat inducible promoter was used to allow for sequential targeting control of the Cas9 activity and the codA was used to enable the selection of “transgene-free” cell lines.

Using the above described “self-removable” vector, the Cas9 activity on different targets can be sequentially controlled. At first, Cas9 is guided, by the constitutively expressed sgRNAs-XylT/FucT to knockout the two plant glycotransferases, xylosyl transferase, and fucosyl transferase. Once the knockout is accomplished and confirmed, a second step is triggered by heat induction in order to express the sgRNA-Z, subsequently targeting the Cas9 to the two ZZZ borders. Dual, simultaneous, DNA digestions, at both sides of the transgenic insert, can result in the excision of the DNA insert from the cell genome, followed by non-homologous end joining (NHEJ) DNA repair (Gorbunova and Levy, 1997, 1999). This chain of events can consequently result in XylT/FucT knockout cells, which are free from foreign DNA.

### Knockout of the XylT and FucT Genes Using the “Self-Removable” CRISPR-Cas9 Vector

**FIGURE 2:**
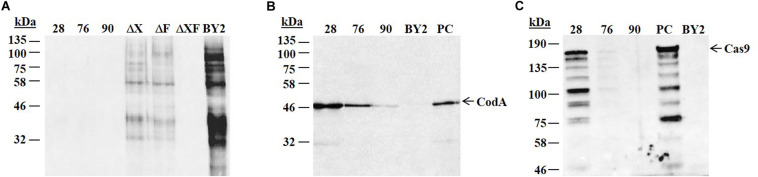
Western blot analysis to characterize putative knocked out lines. Total protein was extracted from the 3 putative knocked out cell lines 28, 76, 90, the non-transgenic BY2 cells and three control knocked out cell lines. A total of 10 μg protein from each sample were loaded on 12% SDS-PAGE followed by western blot using anti-HRP **(A)**, anti-codA **(B),** and anti-Cas9 **(C)** antibodies. kDa: molecular weight in kilo Dalton. Arrowhead indicates the presence of the relevant protein. Expected size of codA is ∼49 kDa. Expected size of Cas9 is ∼159 kDa. **(A)** ΔX, ΔF, and ΔXF represent the knocked out cell lines established previously in our lab (Hanania et al., 2017) and used as controls: ΔX lacks glycans containing Xylose, ΔF lacks glycans containing Fucose, ΔXF lacks glycans containing both Xylose and Fucose. BY2 – positive control (the non-transgenic BY2 cells) that produces Fucose and Xylose containing glycans. **(B)** BY2 represents a negative control (the non-transgenic BY2 cells) and PC represents a positive control (cell line expressing codA). **(C)** PC represents a positive control (cell line expressing Cas9) and BY2 represents a negative control (the non-transgenic BY2 cells).

The above described vector was used to stably transform *N. tabacum* BY2 cells. A total of 100 transformed clones (selected on hygromycin) were isolated. To select for clones which were free of glycans containing Xylose/Fucose the lines were subjected to an ELISA test. Three cell lines, 28, 76, and 90, out of the 100 tested clones, presented a putative knockout of both *XylT/FucT* genes (ΔXF). Further analysis by western blot did not detected these cell lines by the anti-HRP antibodies confirming that these 3 clones were fully devoid of glycans containing Xylose and Fucose (Figure 2A and Supplementary Figure 1A).

In order to activate the self-excision step and to enable the further selection of cells that lost the transgene, the presence of the codA and the Cas9 in lines 28, 76, and 90 was essential. To confirm the expression of the codA and Cas9 proteins in these three selected cell lines, further analysis was done by western blot using anti-codA and anti-Cas9 antibodies. All three lines exhibited codA expression, though at significantly different levels (Figure 2B and Supplementary Figure 1B). Cas9 expression in line 28 was relatively high, while line 76 presented a lower expression level and line 90 did not show any signal of Cas9 (Figure 2C and Supplementary Figure 1C). As Cas9 was not detected in line 90, this cell line was omitted at this point from further evaluation.

### Activating the “Self-Removable” Mechanism for the Excision of the Inserted T-DNA

Once the targeted mutations of the *XylT* and *FucT* genes were accomplished and confirmed, the self-removal mechanism was activated by inducing the expression of the sgRNA-Z. For this, two selected cell lines (lines 28 and 76) were repeatedly heat treated. Following the induction, the cells were cultivated in 5-Flurocytosine (5-FC) supplemented culture medium. Cells expressing codA do not survive in the presence of 5-FC. Therefore, the presence of the 5-FC in the culture medium served as a selection for the cell population, which lost the codA activity. Cells that survived the 5-FC medium, were assumed to have lost the codA coding sequence, either by partial deletion or by excision of the entire recombinant DNA insert(s). Assuming that the Cas9:sgRNA-Z activity resulted in a heterogenic cell population, the post induced cells were labeled as 28-pool and 76-pool, hereafter.

### Assessing Likelihood for Excision Events and Selecting a Cell Pool for Further Analysis

**FIGURE 3:**
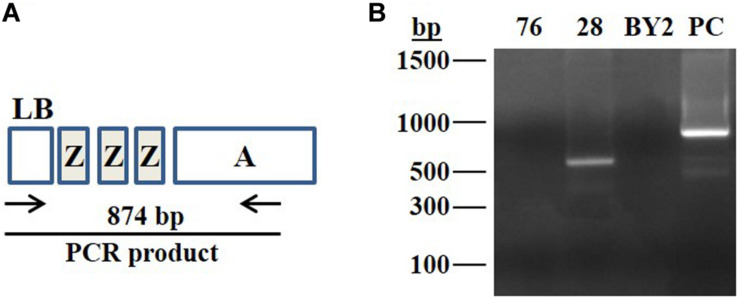
PCR assay to detect functionality of the induced sgRNA-Z:Cas9. **(A)** Schematic illustration of the expected PCR product produced by using specific primers to assess the presence of the *HptII* cassette at the left border of the T-DNA. LB, left border; ZZZ, three repeats of 23 nucleotides each used as a target for gRNA-Z; Cassette A, (NosP-HptII-NosT). The expected size of the PCR product is 874 bp. **(B)** Total genomic DNA extracted from the cell pools of lines 76 and 28, originated from the XylT/FucT knocked out cell lines after repeated heat treatments to induce the sgRNA-Z expression. DNA samples were subjected to PCR analysis. The produced PCR products were separated on 1% agarose gel; BY2 represents negative control (the non-transgenic BY2 cells). PC represents positive control, DNA template produces a 874 bp fragment; bp represents molecular weight marker in base pairs.

In order to detect functionality of Cas9:sgRNA-Z complex, on the ZZZ target, and to assess the probability of occurrence of potential excision events, the 28-pool and 76-pool cells were subjected to PCR analysis using forward primer for the left border of the T-DNA and reverse primer for the *HptII* gene, outside the boundaries of left ZZZ sequence (Figure 3 and Supplementary Table 2 primers 1, 2). These PCR products were generated from a heterogenic pool of cells, thus it was assumed that some products may have been missed by the PCR.

A fragment of 874 bp (illustrated in Figure 3A) is expected to be produced in cells that did not undergo Cas9:sgRNA-Z activity. PCR failure to produce this fragment or any PCR product other than the predicted 874 bp size can indicate endonuclease activity within the left ZZZ repeat sequence.

Using the above described forward and reverse primers, a ∼600 bp fragment was produced with the 28-pool cells and no PCR product was detected in the 76-pool cells (Figure 3B). These results can indicate the occurrence of some mutations within the left ZZZ sequence of the 28-pool and the loss of this sequence within the 76-pool. These results serve as evidence for the actual functionality of the Cas9:sgRNA-Z. The 76-pool was chosen for further analyses, since it demonstrated the highest likelihood for an excision event(s) to have been occurred.

### Subcloning of the 76-Pool and Characterization of the Isolated Clones

**FIGURE 4:**
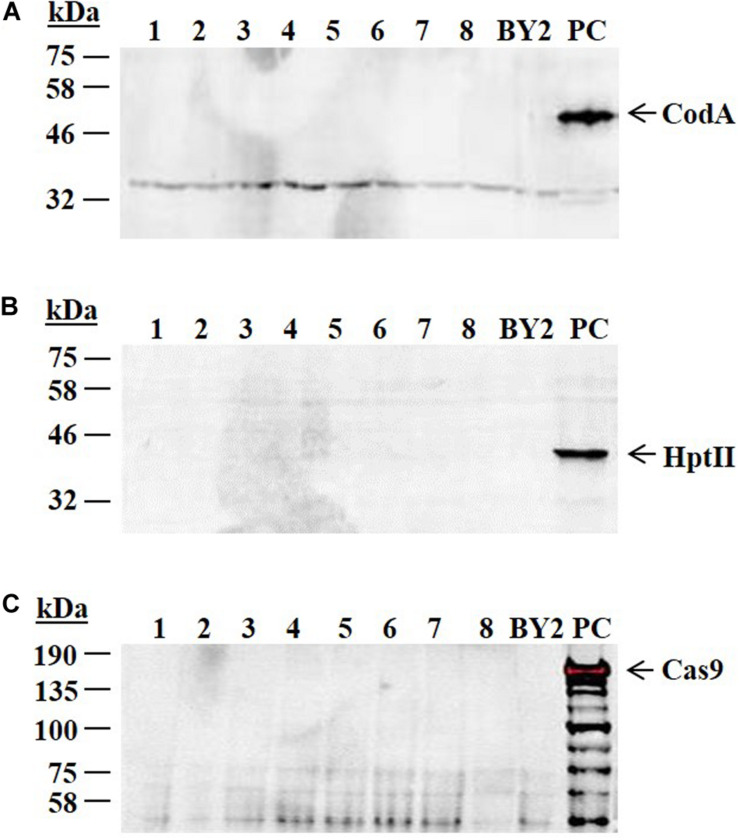
Western blot analysis using anti-codA, anti-HptII and anti-Cas9 antibodies. Eight subclones from the XylT/FucT knocked out 76-pool (subclones 1–8) were analyzed by western blot to detect the presence of codA, HptII, and Cas9 proteins. The amount of 10 μg of total protein from each sample were loaded and proteins were separated on SDS-PAGE followed by western blot using **(A)** anti-codA antibodies **(B)** anti-HptII antibodies **(C)** anti-Cas9 antibodies. PC represents a positive control, a cell line that expresses the codA or HptII or Cas9; BY2 represents a negative control, the non-transgenic BY2 cells; kDa- molecular weight in kilo Dalton. Arrowhead indicates the presence of the relevant protein. Expected size of codA is ∼49 kDa. Expected size of HptII is ∼39 kDa. Expected size of Cas9 is ∼159 kDa.

The cells of the 76-pool that were selected on 5-FC were plated on solid medium and 8 subclones (1–8) were raised and analyzed by western blot analysis. All these clones were negative to anti-codA, anti-HptII and anti-Cas9 antibodies (Figure 4). Moreover, these lines could not grow in the presence of hygromycin.

**FIGURE 5:**
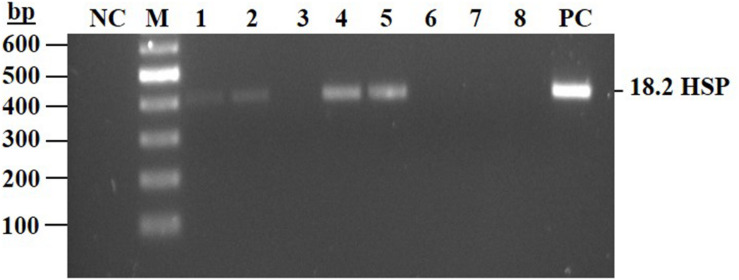
PCR assay to detect the presence of HSP 18.2 promoter in the subclones of line 76 pool. Total genomic DNA was extracted from eight subclones isolated from knockout cell line 76-pool (subclones 1–8). PCR was conducted and products were separated on 2% agarose gel. NC, negative control, mix without template; Lanes 1–8 are isolated subclones derived from the 76-pool; PC, positive control, mix with DNA extracted from transgenic cell line containing the HSP sequence. Expected size for the PCR product 412 bp.

The above selected clones were further analyzed, by PCR, for the presence of the heat shock promoter (*HSP18.2*) sequence. Four subclones 3, 6, 7, and 8 were found negative for *HSP18.2*, while a PCR product was obtained in the other four 76-pool originated subclones (Figure 5 and Supplementary Table 2 primers 4, 5). Two subclones (3 and 7) were chosen for further characterization studies and hence were designated, line 763 and line 767, respectively.

### Confirming Complete Excision by Southern Blot Analysis

Complete excision and the absence of the T-DNA in the genome of lines 763 and 767 were determined by Southern-blot analysis using 4 different probes covering the entire T-DNA at ten different locations. To confirm the absence of a 3.8 kbp section at the 5’ of the T-DNA, genomic DNA was digested with *Pac*I and *Sac*I restriction enzymes and the membrane was hybridized with a DIG *HptII* probe. No signal was detected in these lines (Supplementary Figures 2A,B). To confirm the absence of a 9 kbp section downstream to *HptII* gene, genomic DNA was digested with a single *Nco*I restriction enzyme and the membrane was hybridized with a DIG *hCas9* probe. No signal was detected in these lines (Supplementary Figures 3A,B). To confirm the absence of a 12.4 kbp section downstream to *HptII* gene, genomic DNA was also digested with *Nco*I and *Sph*I restriction enzymes and the membrane was hybridized with a DIG *OcST* probe. No signal was detected in these lines (Supplementary Figures 4A,B). To confirm the absence of the five cassettes of U6-sgRNA, genomic DNA was digested with *Sac*I and *Pac*I restriction enzymes and the membrane was hybridized with a DIG *U6-sgRNA* probe. No signal was detected in these lines (Supplementary Figures 5A,B).

While using primers to the left and to the right borders (Supplementary Table 2 primers 1,3) of the inserted T-DNA (adjacent to the ZZZ sequences), aiming at characterization of the precise sites of the excision, no PCR product was detected. This could be explained by the occurrence of occasional deletions around the break sites. Consequently, we could not determine whether a few bases from the borders of the T-DNA were left at the excision sites.

### Characterization of the Mutations Generated by the Cas9 Multiplexed Targeting of the XylT and the FucT Genes

Cell line 763 was further characterized for the Cas9 generated mutations of the *XylT* and the *FucT* genes. A PCR was performed using 3 sets of primers (Supplementary Table 3) flanking the Cas9 target sites of *XylT* (A and B genes), *FucT* (A, B and C genes), and *FucT* (D and E genes), respectively. The obtained PCR products were cloned into a pGEMT vector and the sub-clones from each sample were sequenced, revealing the presence of assorted insertions and/or deletions (in-dels). No wild type products were detected among any of the tested genes.

Three mutations for the *XylT* genes (Supplementary Figure 6) and seven mutations for the *FucT* genes (Supplementary Figure 7) were identified. An identical 7 bp deletion was found in both alleles of the *XylT-*A gene. A 18 bp deletion and a 7 bp deletions where identified in the *XylT-*B gene. An identical 1,218 bp deletion and 1 bp insertion was found in both alleles of the *FucT*-A gene. A 16 bp deletion in one allele and a 17 bp deletion and 1 bp insertion in the other were found in *FucT*-B. A 1 bp deletion and 1 bp insertion in one allele and 1 bp deletion and 1 bp insertion in the other were found in *FucT*-C.

An identical 1,376 bp deletion and 395 bp insertion was found in both alleles of the *FucT*-D gene and an identical 16 bp deletion and 53 bp insertion was found in both alleles of the *FucT*-E gene.

## Discussion

The main achievement of this study is the development of a stepwise usage of the CRISPR-Cas9 system to allow genome editing followed by inducible self-removal of the integrated T-DNA insert in *N. tabacum* BY2 cells in suspension. This approach allows pre-engineering and improving of cells, to better suit their role as becoming hosts for expressing pharmaceutical proteins’, without limiting their subsequent genetic manipulations. The ability of Cas9 to interact with multiple different sgRNAs thus to target multiple genomic sites as well as the ability to induce transcription of sgRNA by using a HSP, at a desired time, was exploited. To accomplish this goal, we developed a CRISPR-Cas9 based self-removable vector that is sequentially activated to perform a serial set of tasks. This vector can be used in a single transformation procedure for both, editing the cells genome and heat induced removal of the transgene, thereafter.

To the best of our knowledge, no technique that allows the removal of heterologous DNA, specifically, from transformed cells in suspension, has been published. As described in the introduction, previously published methods to avoid integration of unwanted genes, such as using transient expression or transfection of preassembled CRISPR/Cas9/gRNA complex are not efficient in cell cultures. Methods that rely on sexual segregation for the removal of transgenes in whole plants, like repeated back crosses, co transformation of two constructs or homologous recombination using the Cre or FLP recombinases are not applicable in cell suspension as the cells propagate asexually.

Induced auto excision strategy was evaluated in different plants using the Cre gene, under the control of a heat shock-inducible promoter, so that both the marker and Cre genes were excised simultaneously after heat shock treatment (Wang et al., 2005; Cuellar et al., 2006; Khattri et al., 2011; Chong-Perez et al., 2012). This approach resulted in leaky expression of recombinase genes from the HSP (Wang et al., 2005; Cuellar et al., 2006). The influence of this leaky expression on the transformation efficiency in maize lead to nearly a 20-fold reduction (Zhang et al., 2003).

In the current study, we employed an alternative method, using CRISPR-Cas9 to specifically induce DNA double strand breaks (DSBs). These breaks were targeted at two loci, at each of the borders of previously inserted T-DNA, resulting in the removal of the transgene. Contrary to the Cre heat induced auto-excision, in our experiments, the use of the heat shock inducible promoter did not affect the transformation efficiency and the growth level of the cells, in the presence of hygromycin, for an extended period.

We cannot rule out the possibility of leaking activity in our system; however, these results imply that the putative leakiness of the Arabidopsis 18.2 HSP was scanty and not significant in the BY2 cells. Another possible explanation is that the high expression levels of the five sgRNAs targeted to the *XylT* and *FucT* genes successfully competed with the leakiness of sgRNA-Z and thus, the association of the sgRNA-Z with Cas9 was prevented.

We have successfully demonstrated that the application of the developed self-removable vector resulted in *XylT* and *FucT* knockout *N. tabacum* BY2 cells by using constitutively expressed Cas9 and five sgRNAs. In addition, we demonstrated the removal of the 14.3 kbp insert by western blot analyses, using various antibodies, and by Southern blot hybridization, using different probes. The removal step can be activated at any point of time after the cells’ transformation, enabling as much time as required for CRISPR-Cas9 to perform its targeted gene modifications, before its self-excision.

The precise excision site of the T-DNA was not characterized and a few nucleotides of the borders regions might have been left behind. This is not critical, in our case, as our main goal was to obtain a modified platform cell line that is free of the CRISPR-Cas9 and of the selection markers. This host cell line can then be used for the expression of heterologous proteins. This primary goal was successfully achieved.

In tobacco BY2 cells, as well as in other plant cells that are used as host cell lines for the expression of foreign genes, is an obvious advantage to remove the heterologous DNA cassette in order to free the cells from the surplus burden of expression of no longer required heterologous genes. In addition, removing the selection marker enables to conduct further rounds of transformation with another genes of interest using the same selection marker that was used before excision, which prevents the cells from being burdened with additional resistance genes. In this way the necessity to contain a number of unnecessary selection genes is avoided. Furthermore, removing of non-required promoters and genes reduces the chance of gene silencing of a desired transgene that is driven by the same promoters.

Another advantage to excise out the CRISPR-Cas9 system after completing the required modification, is to prevent potential off-target gene modification.

In addition to the application of this method in plant cell cultures, it could also be used to overcome various public and regulatory concerns, like the presence of antibiotic resistance genes in transgenic crop plants.

Since the CRISPR-Cas9 systems have been implemented in numerous organisms, we suggest that our approach can also be applied to diverse genetic systems of other organisms.

## Data Availability Statement

The original contributions presented in the study are included in the article/Supplementary Material, further inquiries can be directed to the corresponding author.

## Author Contributions

MS, UH, and TA conceived the project, designed, and supervised the experiments with the advice of YS. MS designed the constructs and primers. MS and VR performed the DNA extractions, southern blot analysis, all additional molecular biology work, and western blots. AT and DO performed the plant cell transformation, lines selection, and additional cell culture works. UH and TA wrote the first draft of the manuscript. TA and YT contributed to the final editing of the manuscript. All authors read and approved the final manuscript.

## Conflict of Interest

All of the authors were employees of Protalix Biotherapeutics, the sponsor, at the time of this study, and as such have vested commercial interests.
