# Supplementary material for: Sequential Genome Editing and Induced Excision of the Transgene in N. tabacum BY2 Cells
Source: Front Plant Sci. 2020 Nov 25;11:607174. doi: 10.3389/fpls.2020.607174 (PMC7723889; doi:10.3389/fpls.2020.607174)
Supplement: Supplementary file 1 [file Data_Sheet_1.DOCX]

**Supplementary method**. Total genomic DNA extraction

Ten grams of BY2 cells harvested at day 5 were grounded in liquid nitrogen with mortar and pestle. The powdered cells were transferred into 100ml lysis buffer containing 8 M urea, 0.35 M NaCl, 0 .05 M Tris-HCI (pH 7 .5), 0.02 M EDTA, 2% Sarcosyl , 0.3% Sodium thiosulfate and 1% PVP-40 . The mixture was incubated in a 70˚C hot water bath for 60 min. An equal amount of mixed Phenol-Chloroform-Isoamilalcohol (25:24:1) was added, mixed and the phases were separated by centrifugation. Phenol-chloroform extraction was repeated twice. The DNA was sediment after addition of an equal amount of cold Isopropanol, washed with 80% ethanol, dried and immediately dissolved in 1.5 ml TE buffer containing 25 µl of RNaseA (10 µg/ml) and incubated at RT for overnight. An equal amount of mixed Phenol-Chloroform-Isoamilalcohol (25:24:1) was added, mixed and the phases were separated by centrifugation. Chloroform extraction was repeated twice. The phases were separated and an equal amount of Isopropanol was added to the upper phase and centrifuged. Pellet was sediment, washed with 80% ethanol, dried and re-suspended with 400μl TE.
