# Supplementary material for: Sequential Genome Editing and Induced Excision of the Transgene in N. tabacum BY2 Cells
Source: Front Plant Sci. 2020 Nov 25;11:607174. doi: 10.3389/fpls.2020.607174 (PMC7723889; doi:10.3389/fpls.2020.607174)
Supplement: Supplementary file 2 [file Image_1.PDF]

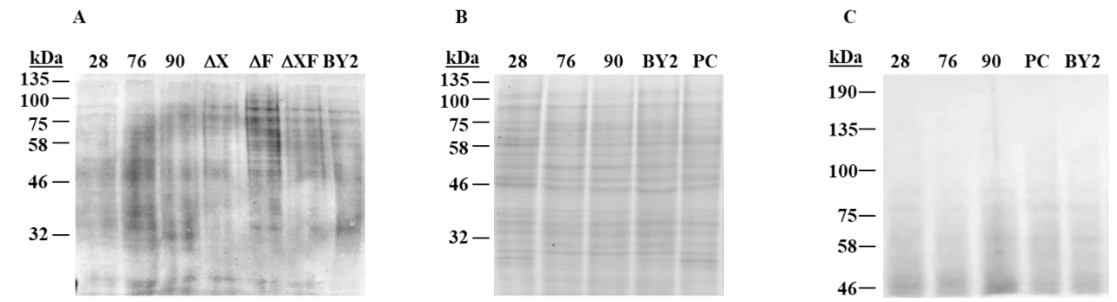


**Supplementary Figure 1.** Ponceau staining of the membranes that were used for western blots in figure 2

Total protein was extracted from the 3 putative knocked out cell lines 28, 76, 90, the non-transgenic BY2 cells and three control knocked out cell lines. A total of 10 µg protein from each sample were loaded on 12% SDS-PAGE followed by western blot using anti-HRP **(A)**, anti-codA **(B)** and anti-Cas9 **(C)** antibodies. kDa: molecular weight in kilo Dalton. Arrowhead indicates the presence of the relevant protein. Expected size of codA is ~49 kDa. Expected size of Cas9 is ~159 kDa. **(A)** ΔX, ΔF, ΔXF represent the knocked out cell lines established previously in our lab (Hanania et al. 2017) and used as controls: ΔX lacks glycans containing Xylose, ΔF lacks glycans containing Fucose, ΔXF lacks glycans containing both Xylose & Fucose respectively. BY2 – positive control (the non-transgenic BY2 cells) that reveals Fucose and Xylose glycans. (**B**) BY2 represents negative control (the non-transgenic BY2 cells) and PC represents positive control (cell line expressing codA). **(C)** PC represents positive control (cell line expressing Cas9) and BY2 represents negative control (the non-transgenic BY2 cells).
