# Supplementary material for: Sequential Genome Editing and Induced Excision of the Transgene in N. tabacum BY2 Cells
Source: Front Plant Sci. 2020 Nov 25;11:607174. doi: 10.3389/fpls.2020.607174 (PMC7723889; doi:10.3389/fpls.2020.607174)
Supplement: Supplementary file 3 [file Image_2.PDF]

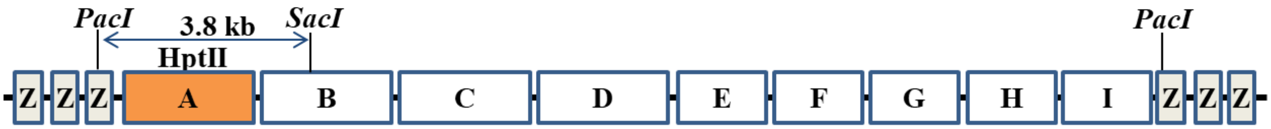

A.

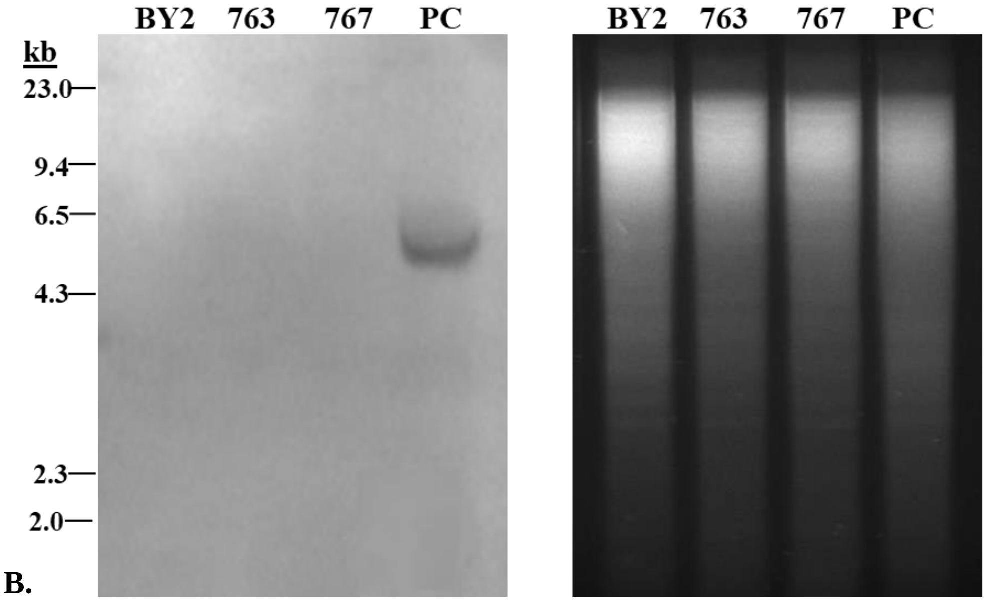

B.

Supplementary Figure 2. Southern blot analysis to confirm excision in lines 763 and 767 using HptII probe

(A) Schematic illustration of the T-DNA integrated into the genome and the HptII probe location (orange). The expected size of the digested *PacI* and *SacI* fragment is 3.8 kb. **(B)** On the right, DNA was separated on 0.8% agarose gel, stained with Ethidium Bromide and then transferred onto nylon membrane. On the left, southern blot analysis of *PacI* and *SacI* digested genomic DNA. BY2 represents cells that are wild type (non-transformed cells), line 763, line 767 and PC represents positive control, transgenic cell line containing the *HptII* gene. The expected size of the positive control digested fragment is 6 kb. Hybridization was done with HptII DNA probe. kb represents DNA molecular weight in kilo-base.
