# Supplementary material for: Sequential Genome Editing and Induced Excision of the Transgene in N. tabacum BY2 Cells
Source: Front Plant Sci. 2020 Nov 25;11:607174. doi: 10.3389/fpls.2020.607174 (PMC7723889; doi:10.3389/fpls.2020.607174)
Supplement: Supplementary file 5 [file Image_4.PDF]

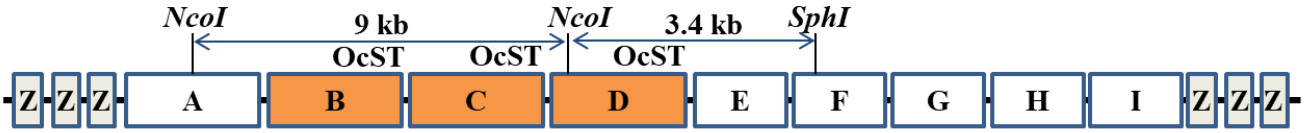


**A.**

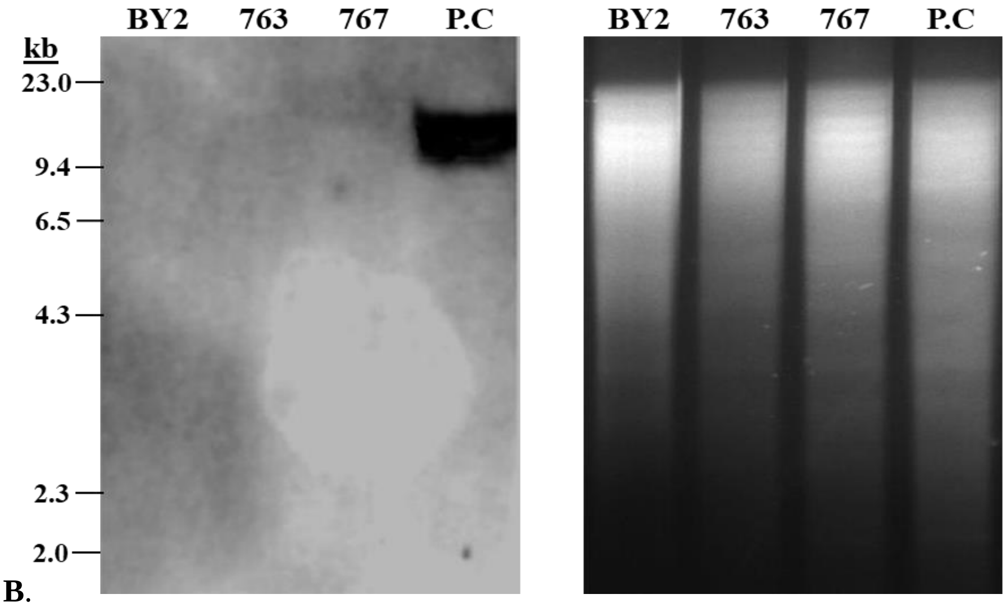


**B.**

**Supplementary Figure 4**. Southern blot analysis to confirm excision in lines 763 and 767 using OcST probe

**(A)** Schematic illustration of the T-DNA integrated into the genome and the OcST probe locations (orange). The expected size of the digested *NcoI* and *SphI* fragments are 9 kb and 3.4 kb. **(B)** On the right, DNA was separated on 0.8% agarose gel, stained with Ethidium Bromide and then transferred onto nylon membrane. On the left, southern blot analysis of *NcoI* and *SphI* digested genomic DNA. BY2 represents cells that are wild type (non-transgenic cells), line 763, line 767 and PC represents positive control, transgenic cell line containing the *OcST* gene. The expected size of the positive control digested fragment is 10.6 kb. Hybridization was done with OcST DNA probe. kb represents DNA molecular weight in kilo-base.
