# Supplementary material for: Sequential Genome Editing and Induced Excision of the Transgene in N. tabacum BY2 Cells
Source: Front Plant Sci. 2020 Nov 25;11:607174. doi: 10.3389/fpls.2020.607174 (PMC7723889; doi:10.3389/fpls.2020.607174)
Supplement: Supplementary file 6 [file Image_5.PDF]

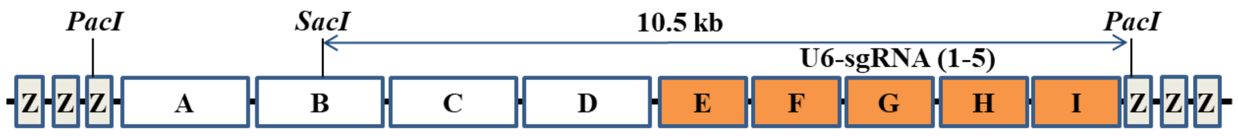

A.

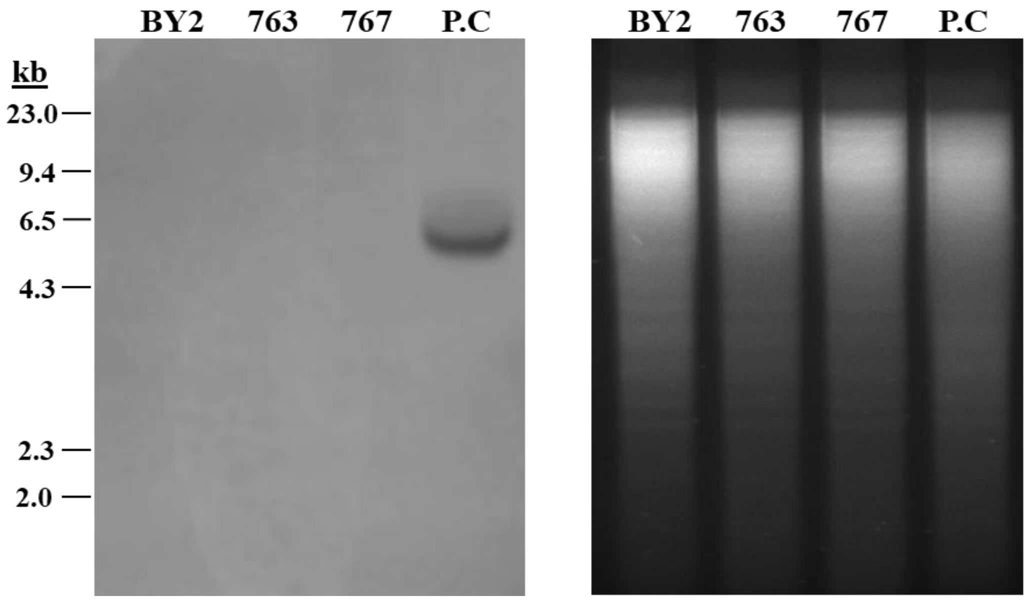

B.

Supplementary Figure 5. Southern blot analysis to confirm excision in lines 763 and 767 using U6-gRNA (1-5) probe

(A) Schematic illustration of the T-DNA integrated into the genome and the U6-gRNA (1-5) probe locations (orange). The expected size of the digested *SacI* and *PacI* fragment is 10.5 kb. **(B)** On the right, DNA was separated on 0.8% agarose gel, stained with Ethidium Bromide and then transferred onto nylon membrane. On the left, southern blot analysis of *SacI* and *PacI* digested genomic DNA. BY2 represents cells that are wild type (non-transgenic cells), line 763, line 767 and PC represents positive control transgenic cell line containing the *U6-gRNA (1-5)* genes. The expected size of the positive control digested fragment is 6 kb. Hybridization was done with mix of U6-gRNA (1-5) probes. Kb represents DNA molecular weight in kilo-base.
