# Supplementary material for: Sequential Genome Editing and Induced Excision of the Transgene in N. tabacum BY2 Cells
Source: Front Plant Sci. 2020 Nov 25;11:607174. doi: 10.3389/fpls.2020.607174 (PMC7723889; doi:10.3389/fpls.2020.607174)
Supplement: Supplementary file 7 [file Image_6.PDF]

```
XylT-A    ATGTCTCGTGGAGGCGAGAAATTGGAGTCGGTTATTGGTAGGAGTGAAGAT
          atgtctcgtggaggcgagaaattggagtcggt-------aggagtgaagat -7

XylT-B    ATGTCTCGTGGAGGTGAGAAATTGGAGTCGGTTATTGGTAGGAGTGAAGAT
Allele 1 atgtctcgtggaggtgagaa------------------taggagtgaagat -18
Allele 2 atgtctcgtggaggtgagaaattggagtcggt-------aggagtgaagat -7
```

Supplementary Figure 6. Sequence analysis of mutations of the *XylT* genes in line 763 Recognition sequences of the *XylT*-A and the *XylT*-B genes are designated in uppercase and sequences of the mutated alleles are shown below in lowercase. One common target site within exon 1, indicated in red, was designed for *XylT*-A and *XylT*-B. Deletions are indicated by dashes and insertions are highlighted in green. The arrow indicates the precise cleavage site. The size of in-del is shown on the right in bp.
