# Supplementary material for: Sequential Genome Editing and Induced Excision of the Transgene in N. tabacum BY2 Cells
Source: Front Plant Sci. 2020 Nov 25;11:607174. doi: 10.3389/fpls.2020.607174 (PMC7723889; doi:10.3389/fpls.2020.607174)
Supplement: Supplementary file 8 [file Image_7.PDF]

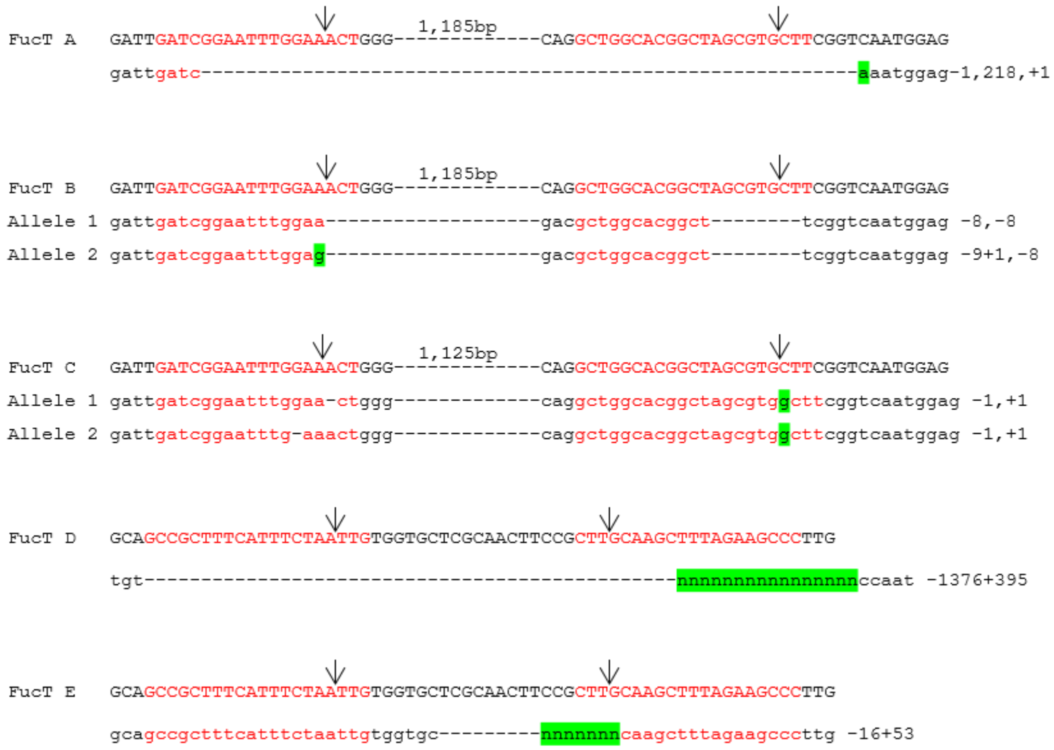

Supplementary Figure 7. Mutations of the *FucT* genes in line 763
Recognition sequences of the five *FucT* genes are designated in uppercase and sequences of the mutated alleles are shown below in lowercase. Two common target sites within exon 1 and exon 2, indicated in red, were designed for *FucT*–A, *FucT*–B and *FucT*–C. Two common target sites within exon 3, indicated in red, were designed for *FucT*–D and *FucT*–E. Deletions are indicated by dashes and insertions are highlighted in green. The arrow indicates the precise cleavage site. The size of in-del is shown on the right in bp.
