# Supplementary material for: Sequential Genome Editing and Induced Excision of the Transgene in N. tabacum BY2 Cells
Source: Front Plant Sci. 2020 Nov 25;11:607174. doi: 10.3389/fpls.2020.607174 (PMC7723889; doi:10.3389/fpls.2020.607174)
Supplement: Supplementary file 9 [file Table_1.DOCX]

**Supplementary Table 1. S**equences of the cas9 target and g-RNA for excision

| Name | Sequence | |
| --- | --- | --- |
| Z | 5’ GCGCTTCAAGGTGCGCATGGAGG 3’ | |
| ZZZ | 5’ GCGCTTCAAGGTGCGCATGGAGGTTAGGCGCGCTTCAAGGT  GCGCATGGAGGGGCGCGCGCTTCAAGGTGCGCATGG 3’ | |
| gRNA-Z | 5’ GCGCTTCAAGGTGCGCATGG 3’ |  |

Three repeats of the Z sequence (ZZZ) were constructed at both boundaries of the ‘self-removable’ vector. An inducible gRNA-Z was included in the vector and once heat shock induced and transcribed, was used to target the Cas9 to the two ZZZ sites at both sides of the integrated insert (T-DNA).
