# Supplementary material for: Sequential Genome Editing and Induced Excision of the Transgene in N. tabacum BY2 Cells
Source: Front Plant Sci. 2020 Nov 25;11:607174. doi: 10.3389/fpls.2020.607174 (PMC7723889; doi:10.3389/fpls.2020.607174)
Supplement: Supplementary file 10 [file Table_2.DOCX]

**Supplementary Table 2.** Primers used to detect excision activity

| # | Name | Sequence | |
| --- | --- | --- | --- |
| 1 | For-LB | 5’ CAGGATATATTGTGGTGTAAACAAATTGACGC 3’ | |
| 2 | Rev- hptII | 5’ GACCGGCTGAAGAACAGCGGGCAGTTCGGTTTC 3’ | |
| 3 | Rev-RB | 5’ CAAACACTGATAGTTTAAACTGAAGGCGGG 3’ | |
| 4 | For-HSP | 5’ CTTTCCATGGTCATTTCTTCTGGTTCAAG 3’ |  |
| 5 | Rev-HSP | 5’ CCTCTTCGAGATACGGGCTCAGTG 3’ |  |

Primers 1,2 - set of primers (Fig. 5) targeted at the left border of the vector and produce a 874 bp DNA fragment (in case of non-excision). PCR failure to produce this fragment can indicate mutations within the left ZZZ sequence. Primers 1, 3- set of primers used to amplify the surplus fragment left between the left and right borders after excision. Primers 4,5 - set of primers (Fig. 7) to test for the present of the heat shock promoter sequence in the cells grew after induction and 5-FC selection. For – forwards; Rev – reverse.
