# Supplementary material for: Sequential Genome Editing and Induced Excision of the Transgene in N. tabacum BY2 Cells
Source: Front Plant Sci. 2020 Nov 25;11:607174. doi: 10.3389/fpls.2020.607174 (PMC7723889; doi:10.3389/fpls.2020.607174)
Supplement: Supplementary file 11 [file Table_3.DOCX]

**Supplementary Table 3.** Primers used for identification and characterization of the mutations in the knocked out cell lines

| # | Name | Sequence |
| --- | --- | --- |
| 1 | For-XylT (A,B) | 5’ CTCTTCGCTCTCAACTCAATCACTCTC 3’ |
| 2 | Rev-XylT (A,B) | 5’ ATTAAYTCACGCATAGTGTGCCTTGAAAT 3’ |
| 3 | For-FucT (A,B,C) | 5’ GAAGGTGTTGGGTCATCATCACCTACAAA 3’ |
| 4 | Rev-FucT (A,B,C) | 5’ TTCTAGATGTGCTTACCCACCGTCGTGC 3’ |
| 5 | For-FucT (D,E) | 5’ GGTTAGTGCTCTTCGTTACATTGAGTCAC 3’ |
| 6 | Rev-FucT (D,E) | 5’ CATCCAGAAAGATGATTTGTCCACAACATT 3’ |

Primers 1,2 - set of primers designed to produce *XylT* (A and B) alleles. Primers 3,4 - set of primers designed to produce *FucT* (A , B and C) alleles. Primers 5,6 - set of primers designed to produce *FucT* (D and E) alleles. For – forwards; Rev – reverse.
